# Atomic-resolution imaging of rutile TiO_2_(110)-(1 × 2) reconstructed surface by non-contact atomic force microscopy

**DOI:** 10.3762/bjnano.11.35

**Published:** 2020-03-10

**Authors:** Daiki Katsube, Shoki Ojima, Eiichi Inami, Masayuki Abe

**Affiliations:** 1 Graduate School of Engineering, Nagaoka University of Technology, 1603-1 Kamitomiokamachi, Nagaoka, Niigata 940-2188, Japan; 2 Graduate School of Engineering Science, Osaka University, 1-3 Machikaneyama, Toyonaka, Osaka 560-8531, Japan; 3 School of Systems Engineering, Kochi University of Technology, 185 Miyanokuchi, Tosayamada, Kami, Kochi 782-8502, Japan

**Keywords:** non-contact atomic force microscopy, (1 × 2) reconstruction, rutile, surface structure, titanium dioxide (TiO_2_)

## Abstract

The structure of the rutile TiO_2_(110)-(1 × 2) reconstructed surface is a phase induced by oxygen reduction. There is ongoing debate about the (1 × 2) reconstruction, because it cannot be clarified whether the (1 × 2) structure is formed over a wide area or only locally using macroscopic analysis methods such as diffraction. We used non-contact atomic force microscopy, scanning tunneling microscopy, and low-energy electron diffraction at room temperature to characterize the surface. Ti_2_O_3_ rows appeared as bright spots in both NC-AFM and STM images observed in the same area. High-resolution NC-AFM images revealed that the rutile TiO_2_(110)-(1 × 2) reconstructed surface is composed of two domains with different types of asymmetric rows.

## Introduction

Titanium dioxide (TiO_2_) is a well-known photocatalyst and has been studied for applications in water splitting and the coating of materials [1]. To optimize the photocatalytic function, it is important to understand the reaction process, hence investigations of chemical and physical surface characteristics and the structure of the photocatalyst are necessary.

The rutile TiO_2_(110) surface has often been the subject of atomic-level studies in the field of photocatalysis since the preparation of a clean surface is relatively easy. A well-known rutile TiO_2_(110) surface is the (1 × 1) structure [2]. The (1 × 1) surface has been studied using low-energy electron diffraction (LEED) [3–4], surface X-ray diffraction [5], non-contact atomic force microscopy (NC-AFM) [6–9], scanning tunneling microscopy (STM) [10–12], transmission electron microscopy [13–14], and density functional theory (DFT) [15–19]. These studies have determined many surface properties such as structure, local defects, and adsorption sites.

**Figure 1:**
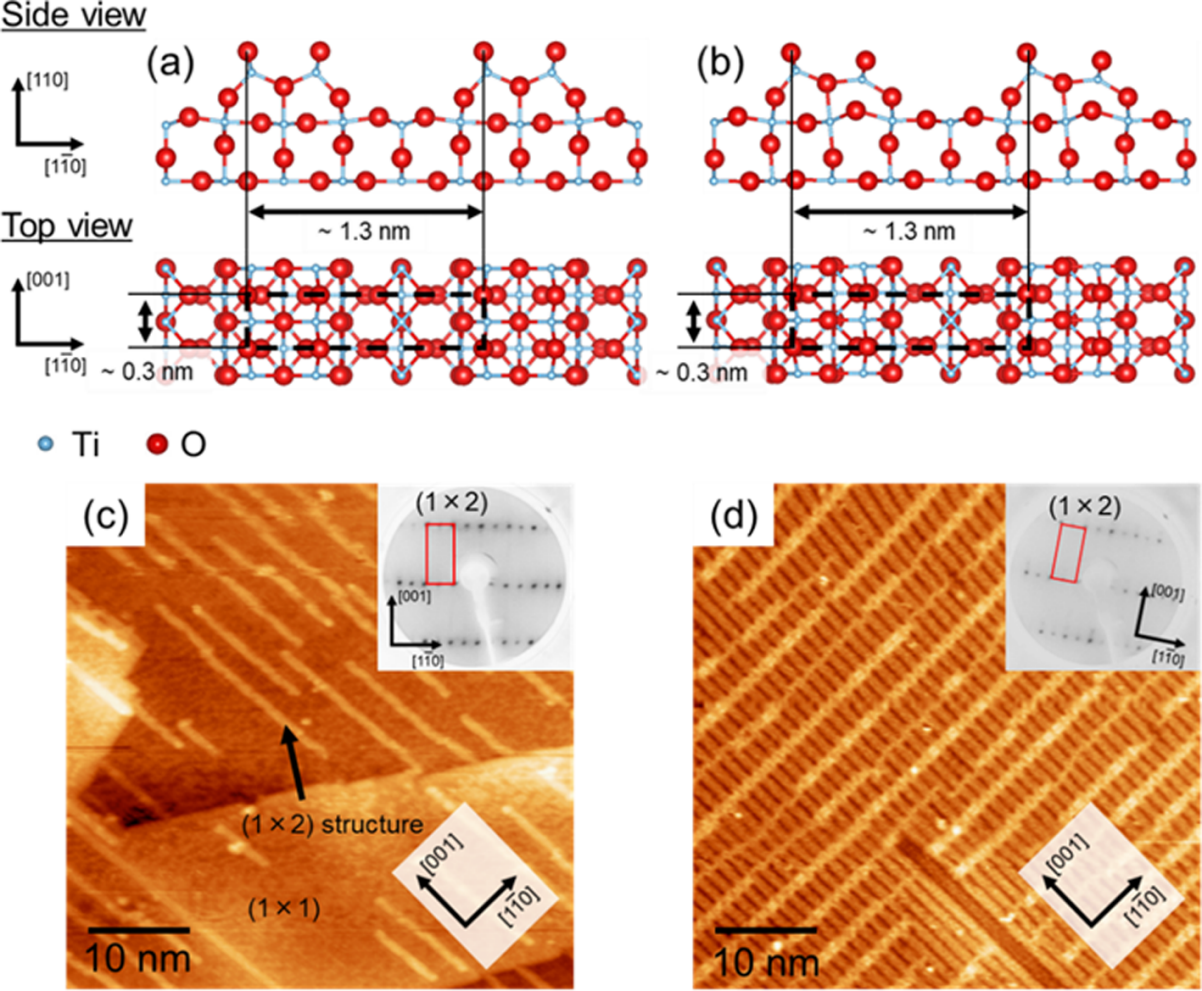
Structural models of rutile TiO_2_(110)-(1 × 2) reconstructed surface: (a) Symmetric Ti_2_O_3_ model [10] and (b) asymmetric Ti_2_O_3_ model [24]. Structural models were made with VESTA [39]. STM images and LEED patterns of the (1 × 2) structure is formed over a local area (c) and a wide area (d). Sample bias voltage and tunneling current were 1.5 V and 10 pA, respectively. LEED patterns were obtained with an energy of 100 eV.

The (1 × 1) surface transforms to the (1 × 2) surface by oxygen reduction in ultra-high vacuum (UHV) [2,20]. Several structural models for the (1 × 2) surface have been proposed [10,21–24]. Onishi and Iwasawa proposed a symmetric Ti_2_O_3_ model (Figure 1a) based on STM measurements [10], while Wang et al. proposed an asymmetric Ti_2_O_3_ model (Figure 1b) similar to the symmetric Ti_2_O_3_ model based on DFT calculations [24]. These two structural models have been widely accepted. Mochizuki et al. reported total reflection high-energy positron diffraction results for the (1 × 2) surface, which supported the asymmetric Ti_2_O_3_ model [25]. In contrast, our previous study using LEED and STM has revealed that the (1 × 2) LEED pattern was observed even if the (1 × 2) structure is formed only partially as shown in Figure 1c [20]. This indicates that real-space imaging with atomic resolution, i.e., STM and NC-AFM, would be helpful for a careful determination of the surface structure. It is necessary to observe the surface directly in order to find out whether the (1 × 2) structure is formed over a wide area. In real-space analysis at the atomic level, simultaneous NC-AFM and STM measurements in UHV at low temperature have revealed that the (1 × 2) chain on the (1 × 1) surface has an asymmetric structure [26]. However, in previous studies on the (1 × 2) surface formed over a wide area of a rutile TiO_2_(110) surface, the periodic line structure of the (1 × 2) surface was considered to be a symmetric structure [10,22,27–28]. Therefore, it is still controversial whether or not the periodic (1 × 2) surface is a symmetric structure. The determination of the surface structure is crucial to understand the surface phenomena, such as adsorption, absorption, and decomposition in photocatalytic reactions.

In this study, we characterized the periodic structure of the rutile TiO_2_(110)-(1 × 2) reconstructed surface using NC-AFM at room temperature. We confirmed by LEED and STM measurements that the (1 × 2) surface forms over a wide area of the rutile TiO_2_(110) surface. Ti_2_O_3_ rows were visualized as bright lines in both STM and NC-AFM images and were observed in the same area. High-resolution NC-AFM imaging revealed that the Ti_2_O_3_ rows are asymmetric structures.

## Experimental

All experiments were conducted using our custom-built system combining NC-AFM, STM, and LEED operated in UHV at room temperature [29]. Nb-doped (0.05 wt %) rutile TiO_2_(110) substrates (Shinkosha Corp.) were used. A rutile TiO_2_(110)-(1 × 2) reconstructed surface was prepared by iterating a surface cleaning process of Ar^+^ sputtering (2 keV, Ar partial pressure of 3.0 × 10^−4^ Pa, ion current of ca. 1.1 µA, 10 min) and annealing (substrate temperature of ca. 1000 °C, 30 min). STM and NC-AFM imaging was performed using Pt-coated Si cantilevers (Budget Sensors, ElectriTAP190G). All cantilevers were cleaned by Ar^+^ sputtering (0.6 keV, Ar partial pressure of 1.0 × 10^−5^ Pa, ion current of 0.05 µA, 5 min) before scanning. STM imaging was performed in constant-current mode without cantilever oscillation. NC-AFM feedback control was applied in frequency-modulation mode [30] with constant amplitude oscillation. The cantilever deflection was detected using an optical interferometer [31]. Since the electrostatic force due to the contact potential difference (CPD) between the tip and sample prevents high-resolution NC-AFM imaging, a bias voltage was applied to the sample to minimize the CPD.

## Results and Discussion

**Figure 2:**
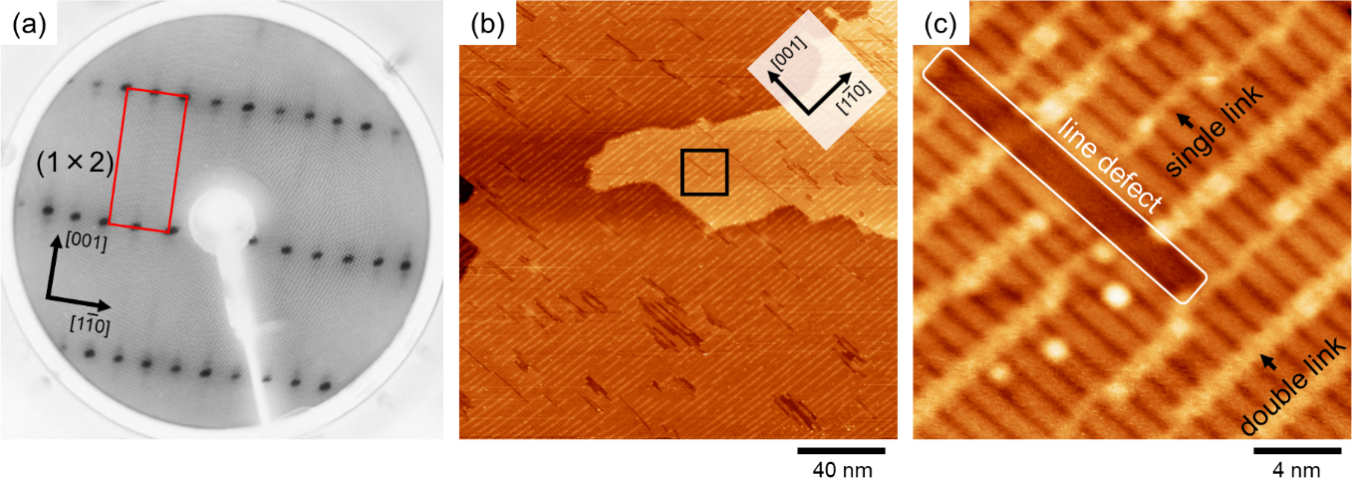
(a) LEED pattern of rutile TiO_2_(110)-(1 × 2) reconstructed surface. The electron beam energy was 100 eV. (b), (c) STM image of rutile TiO_2_(110)-(1 × 2) reconstructed surface (200 × 200 nm^2^ for (b), 20 × 20 nm^2^ for (c)). The sample bias and current set point were 1.5 V and 10 pA, respectively.

Figure 2a shows a LEED pattern of a rutile TiO_2_(110)-(1 × 2) reconstructed surface. The pattern shows two-fold spots in the 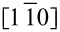 direction, confirming the formation of a (1 × 2) structure. However, in our previous study [20], we reported that a (1 × 2) LEED pattern also appears when the (1 × 2) chain is localized on the (1 × 1) surface. Thus, the surface was observed using STM to confirm that the (1 × 2) reconstructed structure was formed over a wide area of the rutile TiO_2_(110) surface (Figure 2b,c). It can be clearly seen that the (1 × 2) structure was formed over a wide area (200 × 200 nm^2^) of the surface. Some local structures such as single links, double links, and line defects, which have been reported in previous studies [22,27–28,32], are evident on the (1 × 2) surface in Figure 2c. These results confirmed that the (1 × 2) surface prepared in this study is the same surface as in the previous studies [22,27–28,32].

**Figure 3:**
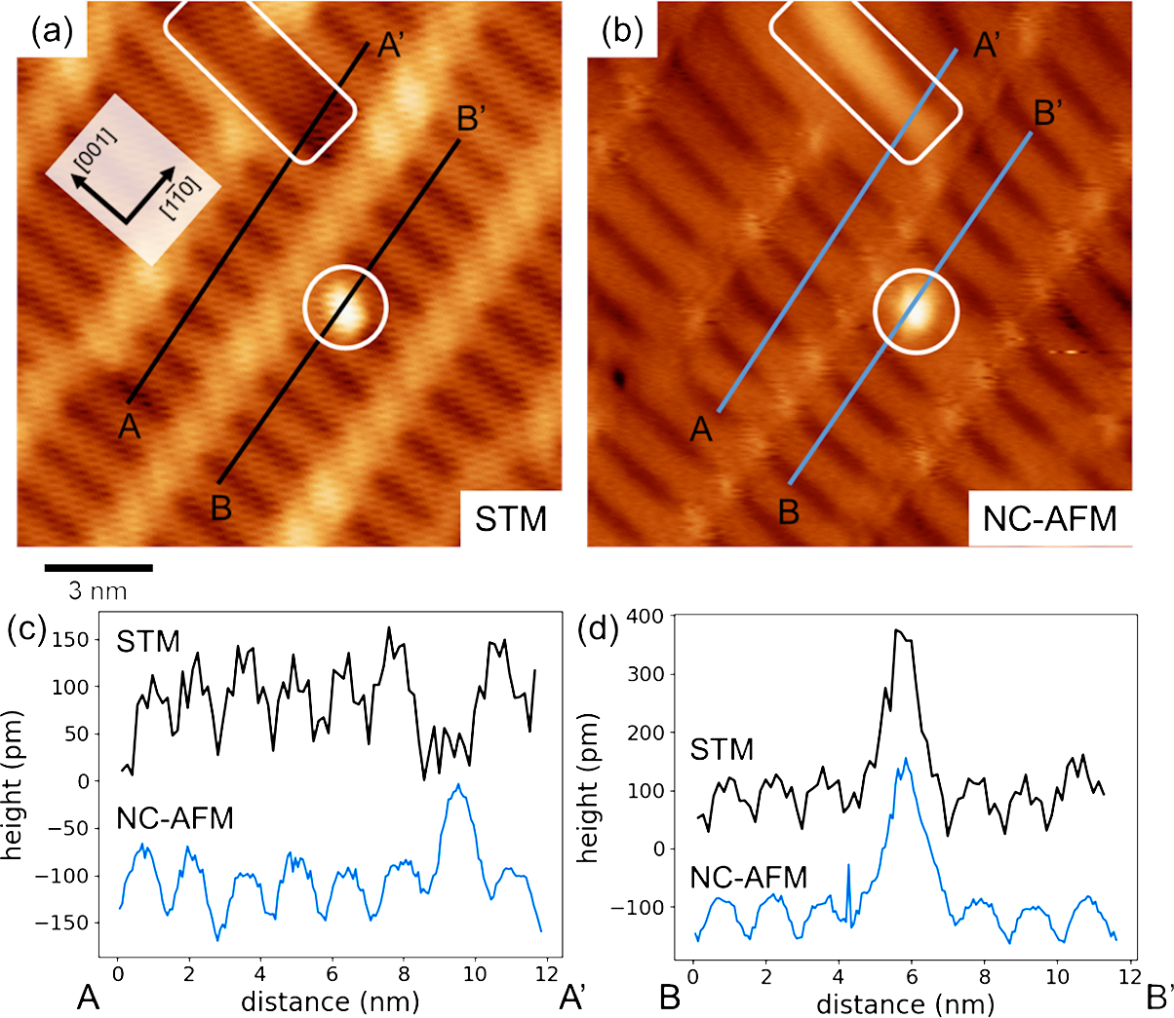
(a) STM and (b) NC-AFM images of a rutile TiO_2_(110)-(1 × 2) reconstructed surface. (c, d) Height profiles along black (STM) and blue (NC-AFM) lines in the images. The STM and NC-AFM images were obtained using a Pt-coated Si cantilever with a resonance frequency of *f*_0_ = 154.1 kHz and a spring constant of *k* = 27.05 N/m. In (a), STM imaging was performed without cantilever oscillation and the parameters sample bias and current set point were 1.5 V and 50 pA, respectively. In (b), the measurement parameters were Δ*f* = −7.9 Hz, *A* = 16.5 nm, and *V*_s_ = 500 mV. The white circles and rectangles in (a) and (b) indicate the same structure at the same position.

Figure 3 shows STM and NC-AFM images and the height profiles obtained from the same surface area. Since STM and NC-AFM use different feedback signals (interaction force for NC-AFM and tunneling current for STM), the surface structure sometimes results in different contrasts in both images. In Figure 3, white squares and circles indicate line defects and protrusions, which are considered to be adsorbates or contamination. A line defect was imaged as a likely vacancy by STM and a protrusion by NC-AFM. By using these defects as markers, the height profiles from STM and AFM along the same lines are compared in Figure 3c and Figure 3d (A–A′ in Figure 3c and B–B′ in Figure 3d, respectively). For each profile, the positional relationship between the periodic lines and the defect is the same. Previous studies have reported STM imaging visualizing Ti_2_O_3_ rows with a bright contrast [22,24,26,28]. Based on these earlier results, the periodic lines with bright contrast in the NC-AFM image can be identified as Ti_2_O_3_ rows. STM and NC-AFM provided different geometry information on the line defect. The line defects could be due to be sub-surface defects because of the geometry of the reflected top surface obtained in NC-AFM imaging using the interaction between the tip and the sample surface as a feedback signal. To identify the line defects, it is necessary to combine DFT and STM and to investigate the bias dependence of simultaneous NC-AFM and STM images. This will be discussed elsewhere since the main subject of this article is the periodic line structure on the rutile TiO_2_(110)-(1 × 2) reconstructed surface.

**Figure 4:**
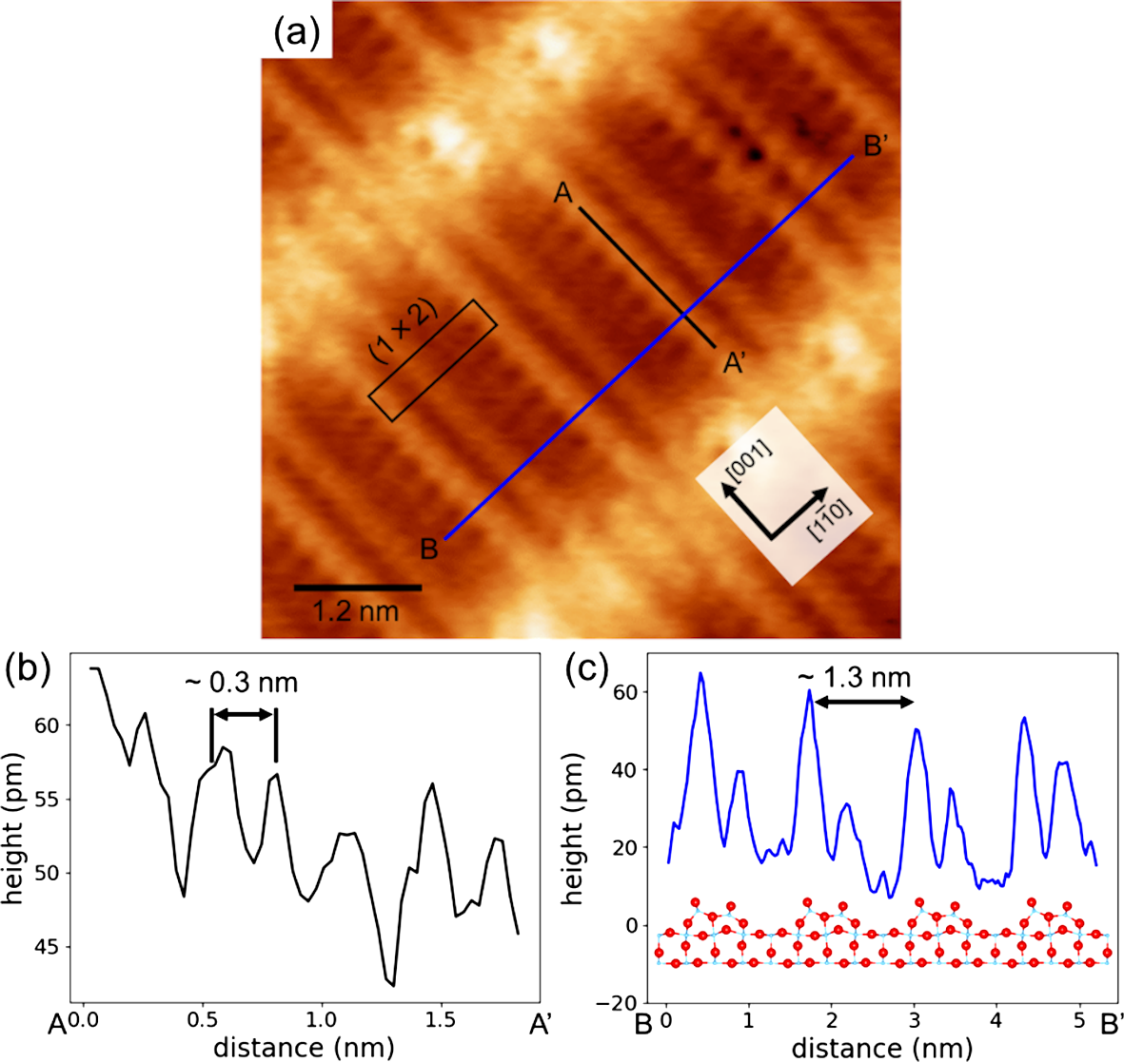
(a) High-resolution NC-AFM image of a rutile TiO_2_(110)-(1 × 2) reconstructed surface. The height profiles in (b) and (c) correspond to the black and blue lines in (a), respectively. The NC-AFM image was obtained using a Pt-coated cantilever with a resonance frequency of *f*_0_ = 154.1 kHz and a spring constant of *k* = 27.05 N/m. The measurement parameters were Δ*f* = −38.1 Hz, *A* = 9.8 nm, and *V*_s_ = 350 mV. The asymmetric Ti_2_O_3_ model is included with the height profile in (d) for comparison of the surface geometry and the model.

Our NC-AFM and STM imaging in the same area identified the Ti_2_O_3_ rows on the rutile TiO_2_(110)-(1 × 2) reconstructed surface. However, the NC-AFM and STM images in Figure 3 could not reveal whether or not the Ti_2_O_3_ rows are symmetric because the tip was too dull to resolve inside the Ti_2_O_3_ rows. To investigate the structure of the Ti_2_O_3_ rows, the rutile TiO_2_(110)-(1 × 2) reconstructed surface was observed with high-resolution NC-AFM imaging using a sharp tip. Figure 4 shows a high-resolution NC-AFM image and height profiles. In the NC-AFM image in Figure 4a, twin resolved Ti_2_O_3_ rows are confirmed. From the height profiles (Figure 4b,c), the pitches of Ti_2_O_3_ in the [001] and 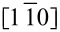 directions were evaluated to be ca. 0.3 nm and ca. 1.3 nm, respectively. These distances correspond to the lattice constant of the (1 × 2) structure. These results confirm that Ti_2_O_3_ rows were observed with atomic resolution. The height profile in Figure 4c shows that Ti_2_O_3_ twin rows are asymmetric, with the left-side rows being higher. These results show that the Ti_2_O_3_ rows of rutile TiO_2_(110)-(1 × 2) reconstructed surface have an asymmetric structure, and thus support the structural model by Wang et al. (Figure 1b) [24].

**Figure 5:**
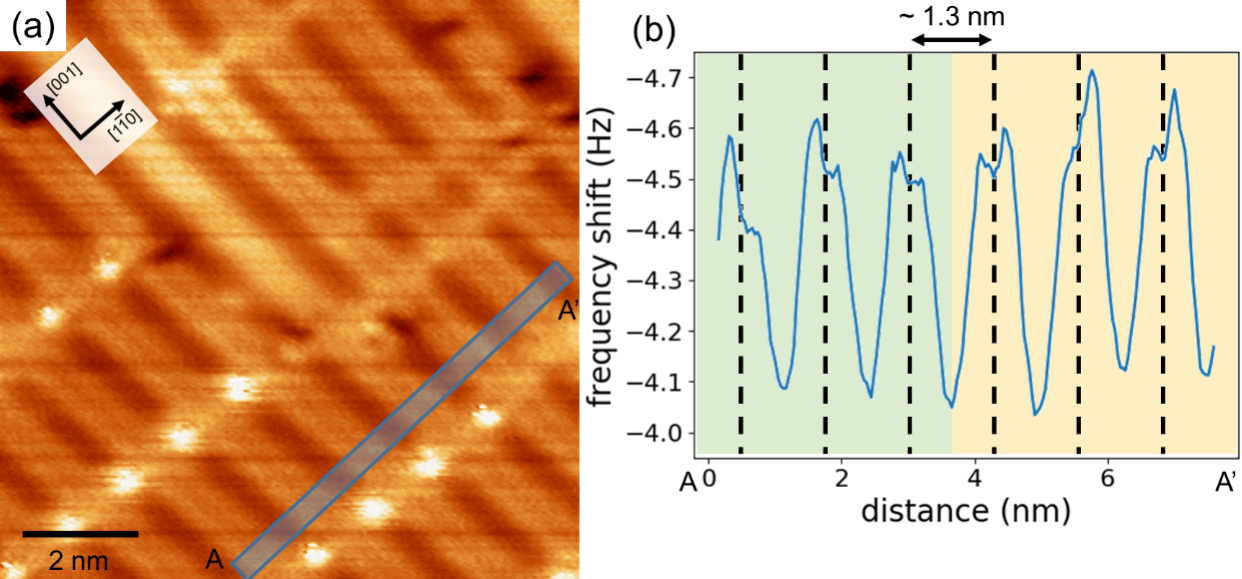
Area exhibiting two types of asymmetric Ti_2_O_3_ rows. (a) High-resolution constant height mode NC-AFM image (raw data) of rutile TiO_2_(110)-(1 × 2) reconstructed surface and (b) averaged line profile corresponding to the blue square in the NC-AFM image. The NC-AFM image was obtained using a Pt-coated cantilever with a resonance frequency of *f*_0_ = 154.9 kHz and a spring constant of *k* = 27.05 N/m. The measurement parameters were *A* = 10.9 nm, and *V*_s_ = 850 mV. The green and yellow regions in (b) indicate Ti_2_O_3_ rows with the left side and the right side in higher positions, respectively.

The image contrast in NC-AFM and STM depends on the structure and the state of the tip apex [7,9,33–34]. Also, deformation of the surface structure sometimes occurs due to interactions between the tip apex and the sample surface [35]. To address the possibility that the asymmetric contrast in the NC-AFM image in Figure 4a is caused by these artifacts, we confirmed the surface asymmetry by changing the AFM tip. By repeating the measurements, NC-AFM images with two types of the different domains were obtained at several times. Figure 5 shows a constant-height mode NC-AFM image (raw data) of a rutile TiO_2_(110)-(1 × 2) reconstructed surface and a height profile. There is a case in which the tip apex asymmetry causes an unexpected local image pattern, i.e., dimers of the same height would be imaged at different heights due to the tip apex asymmetry. We obtained NC-AFM images with two types of asymmetric contrast for during repeated NC-AFM imaging. Two types of Ti_2_O_3_ rows, with either the left side or the right side in a higher position, are shown in the NC-AFM image and height profile in Figure 5, indicating that the asymmetric image is not caused by an asymmetric tip apex structure. The other possibility to be considered is interactions between the tip and the sample surface that cause a deformation of the surface structure. In this case, a non-conservative force induced by surface structure deformations acts between the tip and the sample surface [36–37] and the signal should be observed as an energy dissipation. In the case of surface deformation, a dissipation signal range of 0.3–0.4 eV/cycle on the deformation site has been reported [37–38]. However, our dissipation images showed almost no contrast variation within a range of 0.12 ± 0.11 eV/cycle. This means that the effect of the tip-induced surface deformation is negligibly small, and that the high-resolution NC-AFM images reflect the intrinsic surface structure. These results clearly suggest that the observed asymmetric contrast in the NC-AFM images are attributed to the asymmetric structure of the periodic Ti_2_O_3_ rows. Additionally, the (1 × 2) surface has a multi-domain structure with either the left or the right side in a higher position.

## Conclusion

In summary, we characterized the rutile TiO_2_(110)-(1 × 2) reconstructed surface using NC-AFM, STM, and LEED at room temperature. In NC-AFM imaging, Ti_2_O_3_ rows on the (1 × 2) surface were imaged with high contrast, as confirmed by STM and NC-AFM images obtained in the same area. High-resolution NC-AFM imaging revealed that the Ti_2_O_3_ rows of the rutile TiO_2_(110)-(1 × 2) reconstructed surface have an asymmetric structure. Additionally, we found two domains of asymmetric rows with either the right side or the left side in a higher position. We believe information on the geometry of the rutile TiO_2_(110)-(1 × 2) reconstructed surface is useful for understanding surface phenomena, such as adsorption, absorption, and decomposition in photocatalytic reactions.
